# Disability Severity and Home-Based Care Quality in Older Adults: The Mediating Effects of Social Support and Caregiver Competence

**DOI:** 10.1097/jnr.0000000000000405

**Published:** 2020-09-24

**Authors:** Wei HUANG, Bin HE, Yu Huan WANG, Wen Juan MA, Jia ZHOU

**Affiliations:** 1 BSN, RN, Master Graduate Student, Department of Nursing, Shihezi University School of Medicine, Xinjiang, PRC; 2 MD, Professor and Chief Physician, The People's Hospital of Shihezi City, Xinjiang, PRC; 3 PhD, Professor, Humanities Research Institute, Shihezi City, Xinjiang, PRC.

**Keywords:** structural equation model, disability severity, home-based care quality, Kazakh older adults with disabilities, primary family caregiver

## Abstract

**Background:**

The lack of adequate medical care, healthcare, and older adult care in remote, low-income, rural Kazakh areas of China is a particular concern that should be prioritized for improvement.

**Purpose:**

This study was designed to explore the relationship between the variables of disability severity, social support, and caregiver competence and the quality of home-based care in a population of Kazakh older adults with disabilities and to analyze the path between severity of disability and quality of home-based care in this population.

**Methods:**

A cross-sectional survey was conducted on 335 Kazakh older adults with disabilities living in Xinjiang, China, and their primary informal caregivers. Disability severity was assessed using the Activities of Daily Living Scale, caregiver competence was assessed using the Family Caregiver Task Inventory, social support was assessed using the Social Support Rating Scale, and home-based care quality was assessed using the Family Caregiving Consequences Inventory Scale. Path analysis was used to check the effects of other variables on the quality of home-based care.

**Results:**

Significant correlations were found among disability severity, caregiver competence, social support, and home-based care quality. Disability severity was shown to have a 29.28% direct effect on home-based care quality and a 70.72% indirect effect through social support and caregiver competence.

**Conclusions:**

The results of this study confirm that better social support and caregiver competence improves the quality of home-based care available to older adults with disabilities. Policymakers should give priority to improving the quality of care provided to community-dwelling older adults with severe disabilities. Furthermore, health management departments should provide informal caregiver training that teaches care and rehabilitation knowledge and skills to improve the competencies of caregivers.

## Introduction

Population aging is a worldwide challenge. Eighteen percent of the population in China was 60 years old or older at the end of 2019 (National Bureau of Statistics, PRC, 2020). Moreover, the population of older adults with disabilities has expanded in step with China's aging population. The problems associated with caring for these older adults are becoming increasingly prominent, especially in remote and low-income rural areas of the country, which are disproportionately affected by poverty and poor social service infrastructure. Recent studies have reported that 88.92% of older adults in China's rural areas are willing to receive home-based care, which is currently provided by informal caregivers (Li, 2018). Informal caregivers are primarily children, spouses, and other family members who are not paid for their caregiving services and have poor education and training. Informal caregivers play an indispensable role in the long-term care of older adults with disabilities. However, providing long-term, continuous care leads to physical and mental fatigue in the caregiver, resulting in lower quality of care. Although China has promulgated pilot guidance through the country's long-term care insurance system, this guidance has not been widely implemented (Xia et al., 2018). Meanwhile, the growing population of older adults with disabilities has further increased the care pressures on low-income, rural families and strained public health resources in remote areas.

Xinjiang Uygur Autonomous Region is a multiethnic region in northwestern China, approximately 4,407 kilometers from the capital city of Beijing. Approximately 1.59 million Kazakhs live in the several “Xinjiang pastoral areas.” Medical and healthcare resources are insufficient, and a long-term care system for older adults has yet to be established in the rural and predominantly Kazakh regions of Xinjiang. The nomadic traditions of the Kazakh include ethnic mores that stress providing mutual assistance and support within and among communities (Ali et al., 2013). One study identified the high prevalence of chronic diseases in the Kazakh region of Xinjiang as an important cause of the region's high disability rate among older adults (Maiaiti et al., 2014). Given the low average household income and large size of families in this region, the problems in care faced by older Kazakh adults with disabilities are more urgent than elsewhere (M. Y. Wang et al., 2016). Moreover, Kazakh cultural mores specify that children (sons, daughters-in-law, and daughters) are responsible to care for their parents and that failure to do so must result in condemnation and revilement from other members of the clan (Chen & Chen, 2016). However, long-term care involves a heavy burden in terms of both time and financial resources. Thus, most informal caregivers experience stress and frustration, which reduces the quality of and satisfaction with life for both informal caregivers and care recipients (W. T. Wang et al., 2017).

Home-based care quality has become an especially important issue because older adults typically prefer to receive care in home-based, informal settings rather than in institutions. Home-based care quality includes the degree to which the care recipient's self-perceived care needs are met, the emotional changes and feedback (spiritual support, personal growth) experienced by the caregiver, and the extent to which noncaregiving family members are affected by caregiving (Lin et al., 1998). The family dimension of home-based care quality has received little attention in the literature (Schumacher et al., 1998; Panyavin et al., 2015; Zhou et al., 2019). In one study, there was a negative correlation between severity of disability and quality of home-based care in a population of older adults (Zhou et al., 2019). Caregiver competence directly affects the quality of home-based care (Jiang, 2015) and refers to the ability of home-based primary caregivers to provide appropriate daily care, to seek disease-related knowledge and skills, and to provide mental and emotional support to care recipients (L. X. Wang & Jiang, 2007). However, because home-based caregivers generally lack basic care knowledge and skills, the standards of care fall short of the needs of older care recipients with disabilities, resulting in lower satisfaction with care. Hence, informal caregivers with inadequate caregiver competence require social support to improve care quality. Another study found that older adults with disabilities and chronic diseases require higher than average levels of social support (Burzynska et al., 2016). Social support refers to the objective or subjective influence of various social relationships based on social network institutions on individuals (Yuan & Yao, 2017). Higher levels of social support may not only prevent the health of older individuals from worsening but also improve the quality of home-based care (Chiao et al., 2017).

Improving the quality of care reduces the self-perceived severity of disease in older adults with disabilities and improves emotional function in both older adults with disabilities and their informal caregivers (Panyavin et al., 2015). However, researchers have largely ignored the problems faced by family members and the family as a whole in the context of caring for older adult relatives with disabilities.

On the basis of the previously discussed research, social support and caregiver competence have different degrees of influence on the severity of disability among and quality of home-based care provided to older adults. However, the path of this influence is unclear. Hence, the following two hypotheses are presented in this study: (1) Social support and caregiver competence moderate the causal chain between the severity of disability among and quality of home-based care provided to older adults, and (2) social support and caregiver competence mediate the path relationship between the severity of disability among and quality of home-based care provided to older adults.

In this study, home-based care quality is treated as the outcome variable and the path relationship that affects home-based care quality is clarified via the structural equation model analysis to offer a feasible example for policymakers to improve the quality of home-based care from the perspective of older individuals with disabilities, informal caregivers, and their families. The authors hope that the findings provide a practical reference to improve the quality of home-based care in remote rural areas both in China and in developing countries around the world.

## Methods

### Study Design

This cross-sectional study was conducted from September 2017 to February 2018 in the Xinjiang Uygur Autonomous Region in northwestern China. On the basis of the geographic distribution of the Kazakh population in pastoral areas and the level of per capita disposable income, Yu Min County (low economic level) in Ta Cheng and Fu Hai County (high economic level) in A Le Tai were selected as the geographic targets of this research. Three hundred thirty-five older adults with disabilities and their primary home-based informal caregivers were recruited in the two counties using a multistage, stratified sampling approach (county–countryside–village).

### Participants

Participant–caregiver dyads were recruited from 30 villages in the two target counties. The inclusion criteria for the older adults were as follows: (a) having a disability and 60 years old or older, (b) a Katz ADL score greater than 14 and meeting the standard for disability (light, medium, or heavy), (c) residing in one of the two targeted counties, and (d) providing informed consent to participate. The exclusion criteria were as follows: (a) currently living in a nursing home or planning to enter a nursing home within 6 months and (b) dying within 1 year because of serious illness. The inclusion criteria for the primary informal caregivers were as follows: (a) being a primary (main designated) informal caregiver to the older adult participant and older than 18 years old, (b) providing care without monetary compensation to a family or clan member, (c) providing care to the older adult participant for 6 months or more, and (d) providing informed consent to participate. The exclusion criteria for caregivers were having cognitive impairment, failing to adequately express themselves to the researchers, and refusing to complete the questionnaire.

### Measures

#### Disability severity

The severity of disability among participants was measured using the Katz Activity of Daily Living Scale (ADLS), which was developed by Lawton and Brody in 1969 to evaluate the ability of older adults to perform everyday activities. The Cronbach's alpha and reliability coefficient for this scale were .881 and .84, respectively. The scale included the two dimensions of the Physical Self-Maintenance Scale and the Instrumental Activities of Daily Living Scale, which included six and eight items, respectively. The ADLS was designed to assess degree of ability to perform specific activities using scores ranging from 1 (*totally doable*) to 4 (*completely unable to do alone*); higher scores are associated with a greater degree of disability. A total scale score higher than 14 indicated different degrees of functional decline. A single item score of 1 was normal, whereas scores between 2 and 4 indicated functional decline. The Cronbach's alpha in this study was .928.

#### Caregiver competence

The Family Caregiver Task Inventory (FCTI) was developed by Clark and Rakowski (1983) to assess caregiver competence. The scale consists of five sections and 25 entries. Each entry has three dimensions (no difficulty, difficulty, and extreme difficulty) with dimension scores ranging between 0 and 2 and a total possible inventory score ranging from 0 to 50. The FCTI score indicates the difficulty level of caregiver tasks, with higher scores associated with greater difficulty. The internal consistency coefficient and scale test reliability of the FCTI were previously estimated at .82 and .88, respectively. The Cronbach's alpha in this study was .745.

#### Social support

The Social Support Rating Scale (SSRS) was used in this study to measure the three dimensions of individual social relations. The SSRS includes 10 items (Xiao, 1994) in the three dimensions of objective support, subjective support, and supported utilization, and the total possible score for this scale is 66 points. Total scores below 22 are classified as “low,” those between 23 and 44 are classified as “medium,” and those 45 and higher are classified as “high.” Higher total scores and subscale scores indicate better social support. The Cronbach's alpha for the SSRS was .909 in this study. Because the number of items in each dimension is unequal, to compare the relationships among the dimensions, the three dimensions in this study were separately processed as “standardized” (which means that unequal numbers of items may be compared for different subconcepts).

#### Home-based care quality

The Family Caregiving Consequences Inventory (FCCI), including 21 items in three dimensions (older adult with disability, informal caregiver, and family members), was designed to assess home-based care quality (Shyu et al., 1999). Each item may be scored between 1 (*bad*) and 3 (*good*) based on the requirements of the different contents of care work. The total possible score ranges of the three dimensions are 11–33, 4–12, and 6–18 points, respectively. The total possible FCCI score range is 21–63 points, with higher scores indicating better home-based care quality. In this study, the internal consistency coefficient of this scale was .83, the test–retest reliability was .80, and the Cronbach's alpha coefficient was .811.

### Procedures

Approval for this study from the institutional ethical review board was obtained by the Ethics Committee of the First Affiliated Hospital, Shi He Zi University School of Medicine (registration no.: 2017-004-01). Furthermore, this study was conducted in accordance with the guidelines of standard university hospitals, including informed consent and cooperation, voluntary participation, and anonymity and confidentiality. Written informed consent to participate in this study was obtained from all of the participants.

### Data Analysis

Statistical analysis of the data was completed using IBM SPSS Statistics Version 23.0 and IBM SPSS Amos Version 21.0 (IBM, Inc., Armonk, NY, USA). The mean, standard deviation, frequencies, and percentages of the demographic characteristics and four variables were summarized using descriptive statistical analysis. The correlation coefficients were calculated to analyze the correction relationships between Katz ADLS, SSRS, FCTI, FCCI, and FCCI dimensions. The standardized path coefficients for each pathway were calculated by the maximum likelihood method. The overall model fit degree index value was diagnosed using the chi-square statistic (χ^2^/degrees of freedom [*df*]), root mean square error of approximation, goodness-of-fit index, and comparative fit index (Bagozzi & Yi, 1988). The recommended sample size of the structural equation modeling method was more than 200. The number of samples in this study exceeded that suggested by the structural equation modeling method, which supported the stability of the model used. Disability severity was the independent variable, and home-based care quality was the dependent variable. The structural equation model was used to test whether social support and caregiver competence had a mediating effect on the relationship between disability severity and home-based care quality. Given that the bootstrap method is highly accurate for testing indirect effects, especially in samples with fewer than 400 participants, this method was used in this study to provide evidence of the indirect impact (Preacher & Hayes, 2008). The testing of the mediation hypothesis to calculate the confidence interval (95%) was completed using the deviation correction bootstrap method with 1,000 samples. When the 95% confidence interval does not include zero, the indirect effect is deemed to be prominent. All of the variables in the structural equation model were numerical variables.

## Results

### Demographic Characteristics of the Participants

The mean age of the participants in this study who were older adults with disabilities was 67.59 years. Less than half (*n* = 137, 40.9%) were men, and 198 (59.1%) were women. A large majority (*n* = 299, 89.2%) had an education below the primary school level. As for the participants who were informal caregivers, 70.4% (*n* = 236) were women. Their average age was 39.36 (*SD* = 15.10) years, and 84% were educated to the junior high school level or below. These participants were primarily children of the care recipients (*n* = 252, 75.2%), including sons, daughters-in-law, and daughters. Family monthly income was at or less than US$235.48 for approximately 70% of the dyads, and most informal caregivers (*n* = 272, 81.2%) engaged in part-time work in addition to their caring responsibilities (Table 1).

**Table 1:** Sociodemographic Characteristics of the Participants (*N* = 335)

Characteristic	Older Adults With Disabilities		Informal Caregivers	
	*n*	%	*n*	%
Age (years; *M* and *SD*)	67.59	7.89	39.36	15.10
Gender				
Male	137	40.9	99	29.6
Female	198	59.1	236	70.4
Educational level				
Illiterate	112	33.4	32	9.6
Primary school	187	55.8	97	29.0
Junior school	27	8.1	152	45.4
High school or above	9	2.7	54	16.0
Relationship with the care recipient				
Spouse			78	23.3
Children (son, daughter-in-law, daughter)			252	75.2
Other relative			5	1.5
Family monthly income (US$)				
≤ 235.48			234	69.9
> 235.48			101	30.1
Employment status				
Full-time work			34	10.1
Part-time work			272	81.2
Unemployed			20	6.0
Retired			9	2.7

### Measure Scores

The average score on the Katz ADLS was 27.58 (*SD* = 6.20). Those with mild, moderate, and severe disabilities scored an average of 24.17 (*SD* = 2.77), 31.52 (*SD* = 1.48), and 39.46 (*SD* = 4.38), respectively. The mean score for social support was 32.51 (*SD* = 8.78); the standardized mean scores for objective support, subjective support, and utilization of support were 2.13 (*SD* = 0.72), 4.71 (*SD* = 1.08), and 2.43 (*SD* = 0.90), respectively. Subjective support earned the highest dimension score. The average score for caregiver competence was 15.10 (*SD* = 2.62), with the highest average score earned by the dimension “adjusting life to meet care needs.” Home-based care quality was defined as care quality as perceived by older care recipients with disabilities, informal caregivers, and family members, with each subgroup earning respective mean scores of 23.25 (*SD* = 2.30), 8.63 (*SD* = 1.36), and 10.88 (*SD* = 1.26; Table 2).

**Table 2:** Characteristics of Variables (*N* = 335)

Variable	Older Adults With Disabilities		Informal Caregivers		Other Family Members	
	Mean	*SD*	Mean	*SD*	Mean	*SD*
Katz ADLS	27.58	6.20				
Mild	24.17	2.77				
Moderate	31.52	1.48				
Severe	39.46	4.38				
SSRS			32.51	8.78		
Objective support			2.13	0.72		
Subjective support			4.71	1.08		
Utilization of support			2.43	0.90		
FCTI			15.10	2.62		
Adaptation of the caregiver role			2.75	0.86		
Requirement and assistance			3.08	0.83		
Handling personal emotions			2.48	0.85		
Assess family and community resources			3.38	0.81		
Adjust life to meet care needs			3.41	0.87		
FCCI	23.25	2.30	8.63	1.36	10.88	1.26

***Note.*** Data are expressed by mean (*SD*). The total score of social support is the original data, and that of the dimension is the standardized data. Katz ADLS = Katz Activity of Daily Living Scale; SSRS = Social Support Rating Scale; FCTI = Family Caregiver Task Inventory; FCCI = Family Caregiving Consequences Inventory.

### Correlation Analysis

The Pearson correlation coefficients among the Katz ADLS, social support, caregiver competence, and home-based care quality dimensions are depicted in Table 3. Results affirm a significant correlation on the expected paths of the hypothesis model.

**Table 3:** Correlations Between Variables (*N* = 335)

Characteristic	Katz ADLS	SSRS	FCTI	FCCI	Older Adult FCCI	Caregiver FCCI	Family FCCI
Katz ADLS	1						
SSRS	−.133**	1					
FCTI	.638**	−.228**	1				
FCCI	−.461**	.306**	−.601**	1			
Older adult FCCI	−.348**	.231**	−.454**	.755**	1		
Caregiver FCCI	−.364**	.241**	−.474**	.789**	.596**	1	
Family FCCI	−.405**	.268**	−.527**	.877**	.662**	.692**	1

***Note.*** Katz ADLS = Katz Activity of Daily Living Scale; SSRS = Social Support Rating Scale; FCTI = Family Caregiver Task Inventory; FCCI = Family Caregiving Consequences Inventory.

*p < .05. **p < .01.

### Model Fit Indices

The resultant model fit indices (χ^2^ = 10.379, *df* = 6, χ^2^/*df* = 1.730, *p* = .110, comparative fit index = .994, goodness-of-fit index = .990, and root mean square error of approximation = .047) showed a good fit with the hypothesized model.

### The Effect of Path

The direct and total indirect effects of the path in the model were assessed using bootstrap *SE* (standard error) with a 95% bias-corrected confidence interval. The direct effects of Katz ADLS on FCCI (β = −.135), of Katz ADLS on SSRS (β = −.133), of Katz ADLS on FCTI (β = .619), of SSRS on FCCI (β = .179), of FCTI on FCCI (β = −.474), and of SSRS on FCTI (β = −.146) are shown in Table 4, with all effects meeting statistical significance (*p* < .05, with β representing the standardized path coefficient). The outcomes of the direct (β = −.135) and total (β = −.461) effects affirm a 29.28% primary direct effect of Katz ADLS on FCCI.

**Table 4:** Direct Effects of Variables

Path	β	*SE*	Bias-Corrected 95% CI	*p*
Katz ADLS → FCCI	−.135	0.065	[−0.256, −0.004]	.044
FCTI → FCCI	−.474	0.059	[−0.593, −0.357]	<.001
SSRS → FCCI	.179	0.050	[0.083, 0.280]	.001
Katz ADLS → FCTI	.619	0.041	[0.529, 0.691]	.001
SSRS → FCTI	−.146	0.037	[−0.220, −0.075]	<.001
Katz ADLS → SSRS	−.133	0.053	[−0.239, −0.027]	.014
FCCI → older adult FCCI	.755	0.038	[0.673, 0.821]	.001
FCCI → caregiver FCCI	.789	0.024	[0.737, 0.830]	.001
FCCI → family FCCI	.877	0.026	[0.824, 0.924]	﹤.001

***Note.*** Katz ADLS = Katz Activity of Daily Living; SSRS = Social Support Rating Scale; FCTI = Family Caregiver Task Inventory; FCCI = Family Caregiving Consequences Inventory.

In addition, a direct effect (β = .755, .789, and .877; *p* < .01) was validated between home-based care quality and its three dimensions (older adult with disability FCCI, caregiver FCCI, and family FCCI). In this study, family FCCI was the dimension that affected home-based care quality most significantly. Other variables in the model primarily affected the total home-based care quality through family FCCI.

### Mediating Effect

Mediation analysis was used to assess the effect of Katz ADLS on FCCI through SSRS and FCTI (Table 5). In the main path of ADLS on FCCI, the results of the indirect (β = −.326) and total (β = −.461) effects indicate that a total effect of 70.72% is mediated by SSRS and FCTI. Comparing the indirect (β = .019) with the total effect (β = .638) shows that 2.98% of the total effect of the Katz ADLS on FCTI was mediated by SSRS, whereas comparing the indirect (β = .069) with the total effect (β = .249) shows that 27.71% of the total effect of the Katz SSRS on FCCI was mediated by FCTI.

**Table 5:** Indirect Effects of Variables

Path	β	*SE*	Bias-Corrected 95% CI	*p*
Katz ADLS → older adult FCCI	−.348	0.046	[−0.435, −0.258]	<.001
FCTI → older adult FCCI	−.358	0.048	[−0.458, −0.266]	<.001
SSRS → older adult FCCI	.188	0.041	[0.108, 0.267]	<.001
Katz ADLS → caregiver FCCI	−.364	0.044	[−0.446, −0.273]	<.001
FCTI → caregiver FCCI	−.374	0.049	[−0.474, −0.279]	<.001
SSRS → caregiver FCCI	.196	0.043	[0.113, 0.281]	<.001
Katz ADLS → family FCCI	−.405	0.053	[−0.506, −0.295]	<.001
FCTI → family FCCI	−.416	0.052	[−0.517, −0.310]	<.001
SSRS → family FCCI	.218	0.046	[0.127, 0.307]	<.001
Katz ADLS → FCCI	−.326	0.045	[−0.421, −0.244]	<.001
SSRS → FCCI	.069	0.020	[0.035, 0.113]	<.001
Katz ADLS → FCTI	.019	0.009	[0.005, 0.042]	.007

***Note**.* Katz ADLS = Katz Activity of Daily Living; SSRS = Social Support Rating Scale; FCTI = Family Caregiver Task Inventory; FCCI = Family Caregiving Consequences Inventory.

### Test of the Moderating Effect

After the research variables were centralized, home-based care quality (FCCI) was used as the dependent variable in a regression analysis of the control variables (gender and age), disability severity (Katz ADLS), and caregiver competence (FTCI); disability severity (Katz ADLS) and social support (SSRS); and the interaction terms for, respectively, Katz ADLS × FTCI and Katz ADLS × SSRS. The interaction term for Katz ADLS × FTCI was β = .093, ∆*R*^2^ = .005, *p* = .111, and the interaction term for Katz ADLS × SSRS was β = −.013, ∆*R*^2^ = .000, *p* = .793. Because the results were not statistically significant, Hypothesis 1 was not supported.

## Discussion

This study clarified the paths among disability severity in older adults, social support, caregiver competence, and home-based care quality. The results confirm that disability severity has a negative impact on home-based care quality (Jiang, 2015). Xinjiang pastoral areas are dominated by alpine pastures that are grazed seasonally by ethnic Kazakh herders for approximately 5–6 months a year. The nomadic life of this population is associated with a high-salt and high-fat diet that includes marinated meat, salted dairy products, salty milk tea, and nang. These traditions and dietary habits contribute to the high prevalence of hypertension among Kazakh herders, which affects half of the total herder population (48.88%; You et al., 2017) and 80.6% of older adult herders (J. W. Wang et al., 2019). Hypertension is the main cause of disability and mortality in this population. The per capita life expectancy of Kazakh herders of 62.1 years (Liu et al., 2013) is significantly lower than the national average of 76.2 years in China. In addition, monthly income in large (three to four children) Kazakh rural families is low at US$235.48, which translates roughly US$1.3 per capita per day. This level of income is insufficient to pay for the care needs of older adult family members with disabilities, increasing the economic and care burden borne by the entire family. Furthermore, 33.4% of the older Kazakh adults with disabilities in this study were illiterate, and 84% of their informal caregivers were educated to the junior school level or below. Because of the lack of relevant disease and health knowledge and medication guidance, the “formal” care needs of these dyads are currently unmet, which greatly reduces the quality of home-based care. Furthermore, the unstable emotional state of many older adults with disabilities, who have been plagued by chronic diseases for a long period, makes the relationship with their informal caregiver and family members difficult. Thus, the chronicity and complexity of home-based care were factors identified in this study as negatively affecting home-based care quality.

In addition, several mediating relationships were identified in this structural equation model. Disability severity was shown to influence home-based care quality through social support and caregiver competence. Moreover, disability severity was shown to affect caregiver competence through social support. Caregiver competence played a mediating role between social support and home-based care quality. With regard to the potential causes for the mediating effect of path relationship that affects home-based care quality, home-based care in ethnic Kazakh societies in China is influenced by the deep-rooted concepts of “returning children” (i.e., the first child born after marriage is given to the husband's parents, who raise the child as their own; this child is then expected to care for his or her grandparents in their old age; Dong & Zhao, 2015) and “respecting and loving the elderly” (Chen & Chen, 2016). As part of the unique customs of Kazakh society, Kazakh men are usually in an authoritative position and dominate the family economy, whereas women are mainly responsible for managing the daily affairs of the family. In this study, 70.4% of the informal caregivers were female (daughter or daughter-in-law). Heavy tasks such as providing daily care for their older family members, helping with participation in religious activities, raising children, and doing daily housework are physically and mentally exhausting, especially when caregivers themselves are in poor health. Consequently, caregivers who look after older family members invest significant amounts of time and energy, limiting their time for personal rest and social activities. Individuals who are involved in the long-term care of older family members with disabilities have little chance to go out to participate in traditional activities, and their negative emotions cannot be effectively released, which affects the quality of home-based care. Community development efforts and healthcare services in low-income pastoral areas lag significantly behind other areas in China (W. T. Wang et al., 2016). Residents of these areas who are ill are unable to go to the state-designated village clinic in time for medical treatment because of physical distance, decentralized residence, and higher mobility. An early study reported that 57.7% of herders (nomadic lifestyle) reside more than 5 kilometers away from the nearest medical service point (Rui et al., 2011). Given the lack of medical and health resources in remote and poor areas, formal care services cannot provide sufficient backup support for home-based care, which may affect the quality of care. Furthermore, informal caregivers who provide wholehearted care to older adults with disabilities ensure that recipients are better cared for than either the caregiver or other family members.

The results of path analysis revealed that effective social support and better caregiver competencies have the potential to prevent disability from becoming more severe while somewhat improving home-based care quality. On the basis of the actual situation in pastoral areas, community teams of physicians and nurses should conduct health lectures on basic care knowledge and skills and increase the number of home visits for families in which both the older adults with disabilities and children require care. In addition, government departments should establish mobile hospitals (e.g., tent-based mobile health stations, mobile medical vehicles), and policymakers should pay greater attention to the quality of home-based care received by herdsmen in pastoral areas and actively promote the herdsmen's settlement welfare policy.

### Limitations

The causal relationships among the variables could not be confirmed because of the cross-sectional study design used in this study. Moreover, supporting data are lacking to verify that home-based care quality changes over time, as longitudinal data were not collected. In this study, the family dimension of home-based care quality was obtained by asking the primary informal caregiver. However, the perceptions of informal caregivers on care quality may not represent the viewpoint of the entire family. Thus, research in the future should conduct in-depth interviews with core, noncaregiving members of the family to obtain a more objective view of the quality of home-based care.

### Conclusions

The severity of disability in older adults directly affects the quality of home-based care and has an indirect influence via social support and caregiver competence. These findings indicate that boosting the competency of informal caregivers and providing more social support to older care recipients should mediate the negative emotions of informal caregivers and other family members as well as prevent disease severity from worsening. This study complements the existing literature on the family dimension of home-based care quality and provides a reference for similar countries facing similar situations in low-income regions. Improving the quality of home-based care is a significant and meaningful goal, particularly in regions where mitigation of disability severity in older adults is difficult to achieve because of chronic, systemic issues.
